# Conservation of land plant-specific receptor-like cytoplasmic kinase subfamily XI possessing a unique kinase insert domain

**DOI:** 10.3389/fpls.2023.1117059

**Published:** 2023-02-22

**Authors:** Joseph Yayen, Ching Chan, Ching-Mei Sun, Su-Fen Chiang, Tzyy-Jen Chiou

**Affiliations:** ^1^ Molecular and Biological Agricultural Sciences Program, Taiwan International Graduate Program, Academia Sinica and National Chung Hsing University, Taipei, Taiwan; ^2^ Agricultural Biotechnology Research Center, Academia Sinica, Taipei, Taiwan; ^3^ Graduate Institute of Biotechnology, National Chung Hsing University, Taichung, Taiwan; ^4^ Biotechnology Center, National Chung Hsing University, Taichung, Taiwan

**Keywords:** nuclear localization signal, kinase insert domain, receptor-like kinase (RLK), evolution, intrisically disodered region, land plant adaptation, receptor-like cytoplasmic kinase (RLCK)

## Abstract

The number of genes encoding receptor-like kinases (RLKs) has expanded in the plant lineage. Their expansion has resulted in the emergence of diverse domain architectures that function in signaling cascades related to growth, development, and stress response. In this study, we focused on receptor-like cytoplasmic kinase subfamily XI (RLCK XI) in plants. We discovered an exceptionally long kinase insert domain (KID), averaging 280 amino acids, between subdomains VII and VIII of the conserved protein kinase domain. Using sequence homology search, we identified members of RLCK XI with the unique KID architecture in terrestrial plants, up to a single copy in several hornwort and liverwort species. The KID shows a high propensity for being disordered, resembling the activation segment in the model kinase domain. Several conserved sequence motifs were annotated along the length of the KID. Of note, the KID harbors repetitive nuclear localization signals capable of mediating RLCK XI translocation from the plasma membrane to the nucleus. The possible physiological implication of dual localization of RLCK XI members is discussed. The presence of a KID in RLCK XI represents a unique domain architecture among RLKs specific to land plants.

## Introduction

Protein phosphorylation by protein kinases is a fundamental means of cell signaling across all living organisms (Kobir et al., 2011; Jin and Pawson, 2012; Esser et al., 2016). Protein kinases regulate cellular processes through the catalytic transfer of ƴ-phosphate from ATP to serine, threonine, or tyrosine residues on their target substrates (Taylor and Kornev, 2011). Protein kinases are classified into two groups based on differences in their protein kinase fold, eukaryotic protein kinases (EPKs) with conserved subdomains and atypical protein kinases lacking conserved subdomains (Kanev et al., 2019; Modi and Dunbrack, 2019).

The protein kinase domain of EPKs has a bilobal structure with an average length of 250 amino acids (Taylor et al., 2012). It is comprised of 11 conserved subdomains (I-XI) that fold into an N-terminal and C-terminal lobe (Hanks et al., 1988; Taylor et al., 2012). Subdomains I-IV are part of the N-terminal lobe that anchors and orients ATP. Subdomain V forms the linker region between the two lobes. Subdomains VI-XI are part of the C-terminal lobe that binds substrates and brings them close to ATP for phosphorylation. A key regulatory region known as the activation segment is found within the C-terminal lobe (Johnson et al., 1996; Adams, 2003). The activation segment is flanked by the conserved tripeptide motifs Asp-Phe-Gly (DFG) and Ala-Pro-Glu (APE) of subdomain VII and subdomain VIII, respectively (Nolen et al., 2004). The activation segment does not typically form a stable secondary or tertiary structure (Gogl et al., 2019). In this regard, the activation segment exhibits a high propensity for protein disorder, which is found in sequences referred to as intrinsically disordered regions (IDRs). IDRs are protein segments that do not fold into a recognizable secondary or tertiary structure in their native state (Babu, 2016). As an IDR, the activation segment functions as a flexible region containing phosphorylation sites that can regulate protein kinase activity (Kurotani et al., 2014; Wright and Dyson, 2015).

The activation segment has an average length of 20-35 amino acids (Nolen et al., 2004; Taylor et al., 2012); however, there are a few reports of longer and much less conserved versions of the activation segments, called the kinase insert domains (KIDs), found between subdomain VI and VII in mammalian receptor tyrosine kinases (RTKs) (Locascio and Donoghue, 2013). In RTKs, KIDs can be classified according to their length. The boundaries between the conserved subdomains and the KID have been defined by sequence homology among the members of RTK families (Locascio and Donoghue, 2013). Phosphorylation within the KID of RTKs has been shown to regulate cellular processes, such as mitogenesis and chemotaxis (Locascio and Donoghue, 2013). Much less is known about KIDs in other protein kinase groups.

In plants, the largest proportion of protein kinases belong to a group known as receptor-like kinases (RLKs) that play key roles in growth, development, and stress response (Jamieson et al., 2018; Dievart et al., 2020). RLKs structurally resemble RTKs in having an extracellular domain followed by a single-pass transmembrane region and a C-terminal protein kinase domain (Lehti-Shiu et al., 2009; Jamieson et al., 2018; Jose et al., 2020). The closest homologs of RLKs in animals are Pelle and interleukin receptor-associated kinase (IRAK) (Shiu and Bleecker, 2001). However, RLKs form a monophyletic family distinct from other groups of protein kinases but closely related to Rapidly Accelerated Fibrosarcoma (Raf) kinases and RTKs from animals (Shiu and Bleecker, 2001). The number of RLKs has dramatically expanded in plants from as few as one member in the chlorophyte *Ulva mutabilis*, to more than 610 members in *Arabidopsis thaliana* and more than 1,000 copies in *Oryza. sativa* (Shiu et al., 2004; Dievart et al., 2020). Their expansion has led to diverse protein domains interacting with a wide range of ligands across multiple signaling pathways (Dievart et al., 2020; Gong and Han, 2021).

RLKs can be classified based on the presence of conserved domains in their sequences. Those with one or several extracellular ligand-binding domains are called receptor kinases (RKs), and those without are called receptor-like cytoplasmic kinases (RLCKs) (Yamaguchi et al., 2013; Liang and Zhou, 2018). There are 50 RLK subfamilies based on the sequence similarity of their protein kinase domain (Shiu and Bleecker, 2001). The largest subfamilies contain multiple leucine-rich repeats (LRR) domains, which interact with ligands involved in developmental and stress response pathways (Man et al., 2020). For example, CLAVATA1 (CLV1) controls meristem development (Hazak and Hardtke, 2016). Flagellin-sensitive 2 (FLS2) and BRASSINOSTEROID INSENSITIVE 1-associated kinase 1 (BAK1) undergo conformational changes following signal perception that leads to heterodimerization and reciprocal phosphorylation during pathogen perception (Schulze et al., 2010; Sun et al., 2013; Oh et al., 2018).

RLCKs work in tandem with RKs to transduce signals perceived at the plasma membrane and regulate intracellular processes involving fellow RLCKs (Liang and Zhou, 2018). One of the best-characterized plant RLCKs is the BOTRYTIS-INDUCED KINASE 1 (BIK1), which transmits signals upon the perception of microbe-associated molecular patterns by FLS2/BAK1 kinases at the cell surface to downstream intracellular events (Lu et al., 2010; Ma et al., 2020). RLCKs also act as decoys that interact with effectors to reduce pathogen severity and trigger downstream immune response pathways (van der Hoorn and Kamoun, 2008; Paulus and van der Hoorn, 2018).

Plant RLKs have a diverse collection of domain architectures that dictate their function in several signaling pathways (Jose et al., 2020). In this study, we characterize one RLCK subfamily, RLCK XI. We showed that RLCK XI is conserved in land plants and identified a previously unannotated region, referred to as the RLCK XI-KID, which splits the conserved protein kinase domain into two parts. The RLCK XI-KIDs have an average length of 280 amino acids. The RLCK XI-KID is located between protein kinase subdomains VII and VIII, making the KID equivalent to the activation segment in canonical protein kinases. Conventionally, the activation segments have no well-defined tertiary structure and display intrinsically disordered properties, which was also observed in RLCK XI-KIDs. Notably, we observed repetitive nuclear localization motifs in the KIDs, which were functional in mediating nuclear translocation. The full-length RLCK XI showed both plasma membrane and nuclear localization. However, deletion of the transmembrane domain leads to complete nuclear localization. Our study presents evidence of a newfound domain architecture in the RLCK XI members with a unique KID. Furthermore, functional nuclear localization signals were embedded within the KID of RLCK XI members, which might be implicated in the signaling of various kinase cascades.

## Materials and methods

### Sequence retrieval

Protein sequences were gathered from the Joint Genome Institute (JGI) Phytozome 12 database (https://phytozome-next.jgi.doe.gov) unless specified otherwise (Goodstein et al., 2012). Sequences from *Antheros agrestis*, *Antheros angustus*, and *Antheros punctatus* were gathered from the hornwort sequence database (https://www.hornworts.uzh.ch/en/download.html) of the Szovenyi group. Sequences from *Salvinia cucullate* were gathered from the Fernbase (https://www.fernbase.org/). Sequences from *Picea abies* were gathered from the gymnosperm PLAZA plant comparative genomics database (https://bioinformatics.psb.ugent.be/plaza) (Proost et al., 2009).

### Identification of RLCK XI candidates

RLCK XI candidates were identified using the hmmsearch program from the HMMER3 package (Eddy, 2009). The hmmsearch was conducted on 60 proteomes (**Supplementary Table 1**) across the plant lineage against an RLCK XI profile Hidden Markov Model (HMM). The RLCK XI profile HMM comprised the four known members of RLCK XI from *A. thaliana*. The resulting 152 sequences that passed an E-value threshold of 0.01 were classified as RLCK XI candidates (**Supplementary Table 2**).

### Sequence analysis and annotation for domains and motifs

RLCK XI candidates were annotated for domains using the Conserved Domain Search Tool (https://www.ncbi.nlm.nih.gov/Structure/bwrpsb/bwrpsb.cgi) (Lu et al., 2020). Nuclear localization signals within RLCK XI sequences were identified using the Eukaryotic Linear Motif (ELM) resource (Kumar et al., 2022). Conserved motifs were mapped using the Multiple Em for Motif Elicitation (MEME) online toolbox (http://meme-suite.org/) (Bailey et al., 2015). Transmembrane regions were annotated using Deep Transmembrane Hidden Markov Model (DeepTMHMM) (https://dtu.biolib.com/app/DeepTMHMM/) (Hallgren et al., 2022). Intrinsically disordered regions (IDRs) were analyzed using the IUPred2A program configured to the default (long) setting (Erdos and Dosztanyi, 2020). Sequence logos were built using WebLogo (https://weblogo.berkeley.edu/logo.cgi) (Crooks et al., 2004).

### Sequence alignment and phylogenetic analysis

**Figure 1:**
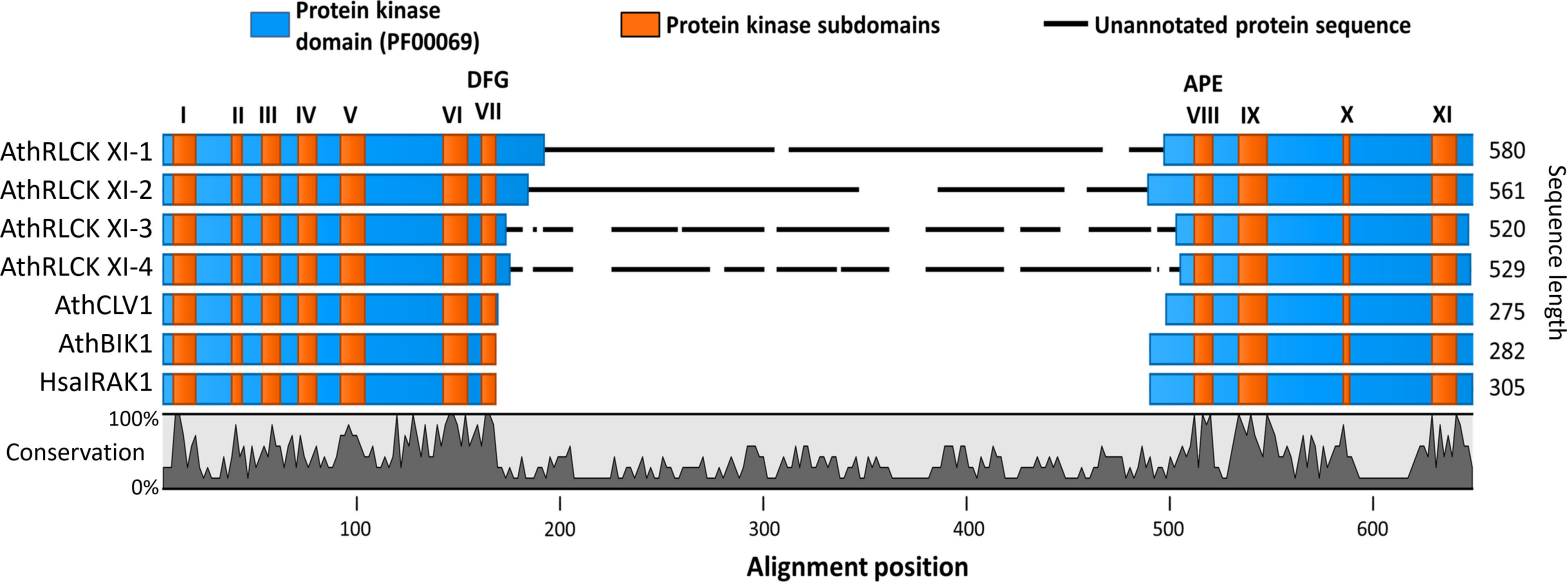
The members of receptor-like cytoplasmic kinase subfamily XI from *A. thaliana* possess a kinase insert domain. Sequence alignment of the protein kinase domains of RLCK XI members, AthRLCK XI-1 (AT1G80870), AthRLCK XI-2 (AT2G45590), AthRLCK XI-3 (AT4G25390), AthRLCK XI-4 (AT5G51770), together with representative RLKs found in A. thaliana. CLV1 (AT1G75820) represents AthLRR-RLK XI-1, BIK1 (AT2G39660) represents AthRLCK VIIa-2, and HasIRAK1 represents RLK found in mammals. The location of conserved protein kinase subdomains (I-XI) on each sequence (orange) was based on sequence alignment with well-characterized protein kinase sequences. An insertion domain in the RLCK XI members was identified between the conserved motifs Asp-Phe-Gly (DFG) and Ala-Pro-Glu (APE) of subdomain VII and subdomain VIII, respectively.

For protein alignment of AthRLCK XI and representative RLKs in **Figure 1**, we aligned members of RLCK XI from *A. thaliana* together with well-studied RLK AthCLV1 from LRR-RLK XI-1 subfamily, AthBIK1 from RLCK VIIa-2 subfamily, and the distant RLK homolog HsaIRAK1 from humans. For **Supplementary Figure 1**, we used the RLK dataset from Dievart et al. (2020), which included grouped subfamilies of RLKs from the iTAK database of protein kinases (http://itak.feilab.net/cgi-bin/itak/index.cgi). To identify suitable RLK representatives, each RLK subfamily was aligned with AthRLCK XI-2. The sequence with the highest pairwise comparison score with AthRLCK XI-2 was chosen to represent their subfamily. AthRLCK XI-2 was selected because it has the highest average pairwise similarity score among all AthRLCK XI members. Each RLK representative selected for alignment contained all eleven conserved protein kinase subdomains. RLK subfamilies whose members lacked one or more conserved protein kinase subdomains were excluded from the alignment. To determine the evolutionary conservation of the RLCK XI subfamily in plants, full RLCK XI protein sequences were aligned using the L-INS-i strategy from MAFFT version 7 (Katoh and Standley, 2013). The resulting alignment was used to construct a phylogenetic tree using the maximum likelihood method from IQ-TREE (Nguyen et al., 2015). The Phylogenetic tree was illustrated using iTOL (https://itol.embl.de) (Letunic and Bork, 2021).

### BLAST search for KID, KID-motif, and NLS-containing sequences

The KID, M2, M3, and NLS sequences found in AthRLCK XI-2 were used as queries for BLAST search against the NCBI non-redundant (nr) protein database (Altschul et al., 2005). The BLAST search was performed using the BLASTP algorithm. The E-value threshold was set to 1 to broaden the coverage of the database search due to the lack of prior knowledge involving sequences containing the RLCK XI-KID.

### Subcellular localization of RLCK XI from *Arabidopsis thaliana* and rice

Full-length protein-coding sequences of AthRLCK XI members and different individual domains were PCR amplified, subcloned into pCR8/GW/TOPO vector, and subsequently recombined into the binary vector by LR reaction following the manufacturer’s instructions (constructs are listed in **Supplementary Table 3**). Next, the constructs were transfected into Arabidopsis protoplasts by polyethylene glycol for transient expression (Yoo et al., 2007). Sixteen hours later, transfected cells were imaged using a Zeiss LSM 780 confocal microscope (Germany). Image analysis was performed with LSM software ZEN.

### Inducible expression of AthRLCK XI in Arabidopsis

The full-length protein-coding sequences of AthRLCK XI-2 and AthRLCK XI-3 with the GFP tag at the C-terminal end were cloned into pMDC7 vector (Curtis and Grossniklaus, 2003) and transformed into *A. thaliana* Columbia (Col-0) accession for inducible expression by estradiol. Transgenic plants were generated through an Agrobacterium tumefaciens (GV3101) dipping procedure (Clough and Bent, 1998). The successful transgenic T2 lines were grown on agar plates with half-strength modified Hoagland nutrient solution (Chiou et al., 2006). Ten-day-old seedlings were transferred to the same medium containing 10 μM β-estradiol. The expression of GFP-tagged AthRLCK XI-2 and AthRLCK XI-3 in the root was observed two days after β-estradiol treatment. The confocal microscopy images of roots were taken using an Andor Dragonfly spinning disk confocal microscope with an objective lens PL APO 40X/1.10 W CORR. Excitation/emission wavelengths were 488 nm/500–550 nm for GFP.

### Expression profile of RLCK XI from *Arabidopsis thaliana*

Publicly available RNA-seq data were obtained from the Arabidopsis RNA-seq database (ARS) (Zhang et al., 2020) (http://ipf.sustech.edu.cn/pub/athrna/). Data from Arabidopsis tissues and biotic stress response were collected for RLCK XI members from *A. thaliana*.

## Results

### Members of RLCK XI from *Arabidopsis thaliana* possess a kinase insert domain

Receptor-like kinases possess a well-defined protein kinase domain that has a length of 250-300 amino acids and includes eleven conserved subdomains (I-XI) (Hanks and Hunter, 1995; Shiu and Bleecker, 2001; Lehti-Shiu and Shiu, 2012). There are over 600 RLKs found in the model organism *Arabidopsis thaliana* that can be classified into more than 50 subfamilies (Shiu and Bleecker, 2001; Shiu and Bleecker, 2003; Dievart et al., 2020). However, only a handful of these RLKs have been studied (Fan et al., 2018; Dievart et al., 2020). Among the RLKs found in *A. thaliana*, we noticed that the four members of RLCK XI, namely, AthRLCK XI-1 (AT1G80870), AthRLCK XI-2 (AT2G45590), AthRLCK XI-3 (AT4G25390), and AthRLCK XI-4 (AT5G51770), had a longer protein sequence compared to other RLCKs by an average of 280 amino acids. We then investigated the origin of the difference in sequence length by aligning the protein kinase domain of AthRLCK XI members with well-studied RLKs AthCLV1, AthBIK1, and HsaIRAK1 to represent the different structural classifications of RLKs (Shiu and Bleecker, 2001; Dievart et al., 2020). The alignment revealed an unannotated region of low similarity that only aligned within members of RLCK XI (**Figure 1**). Closer inspection showed that this region resided in the activation segment between the conserved subdomain VII and VIII of the protein kinase domain. The unannotated region within the protein kinase domain of RLCK XI-1 to 4 had an average length of 276 amino acids, compared to the 20-35 amino acids in the activation segment of AthCLV1, BIK1, and HsaIRAK1 in the same region. We refer to this region as the RLCK XI kinase insert domain (RLCK XI-KID). To reinforce the uniqueness of the RLCK XI-KID among RLKs, we performed additional multiple sequence alignment with representatives from several RLK and RLCK subfamilies from *A. thaliana*. The resulting alignment revealed that none of these other RLCK or RLK subfamilies in *A. thaliana* had sequences similar to the KID (**Supplementary Figure 1**). These results suggest that members of AthRLCK XI possess a unique kinase insert domain located between conserved subdomain VII and VIII within their protein kinase domain.

### The RLCK XI subfamily is conserved in land plants

**Figure 2:**
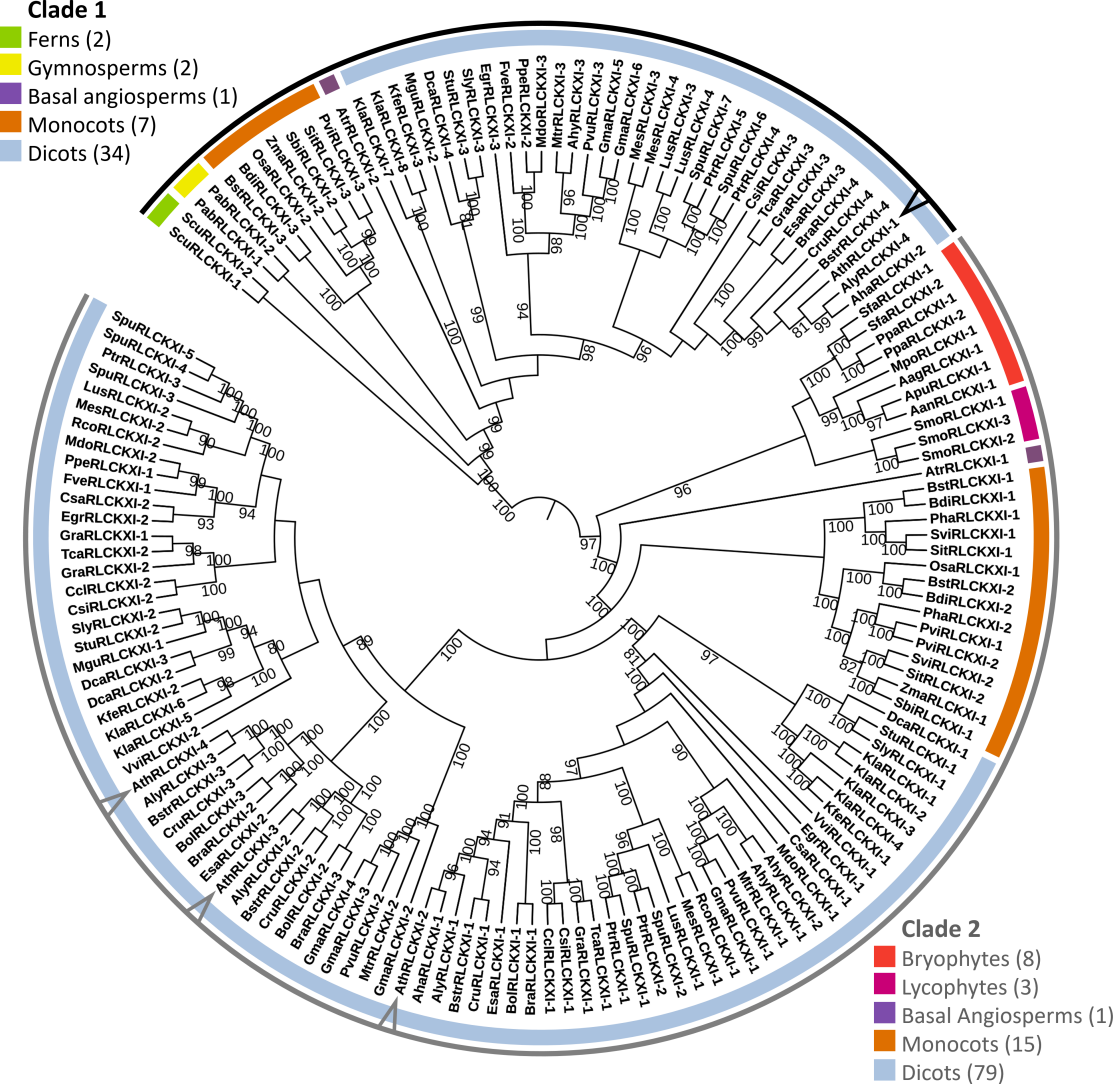
Receptor-like cytoplasmic kinase subfamily XI is conserved in land plants. The phylogenetic tree was constructed based on the protein sequence alignment of 152 full-length RLCK XI candidates from land plants. The alignment was used as input for IQ-TREE with the maximum-likelihood method. The tree is divided into two major clades (Clades 1 and 2), each containing members from different plant lineages represented by different colors. Members from *A. thaliana* are pointed at in the cladogram. Bootstrap replicates were set to 1000. Nodes showing bootstrap confidence of 80 or higher are shown. Visualization and annotation were conducted using iTOL.

To understand whether the members of AthRLCK XI are conserved, we built a profile Hidden Markov Model (HMM) using AthRLCK XI members to search the Phytozome 12 database for potential homologs in the plant lineage (**Supplementary Table 1**). A total of 152 RLCK XI candidate sequences were retrieved using the hmmsearch program of the HMMER3 package (**Supplementary Table 2**). Similar to the naming convention of AthRLCKs, these RLCK XI candidates were assigned names based on their physical locations on the genome of their respective plant species. Annotation of individual protein sequences revealed that all RLCK XI candidates possessed the same domain architecture as those seen in AthRLCK XI members. These results show that RLCK XI is evolutionarily conserved in land plants (**Figure 2**).

The tree was divided into two major clades. Clade 1 consisted of members from ferns, gymnosperms, basal angiosperms, monocots, and dicots. On the other hand, clade 2 consisted of bryophytes, lycophytes, a basal angiosperm, monocots, and dicots. We identified eight RLCK XI members from the bryophytes *Anthoceros agrestis*, *Anthoceros angustus*, *Anthoceros punctatus*, *Marchantia polymorpha*, *Physcomitrella patens*, and *Sphagnum fallax*. The number of RLCK XI members increased from a single member in bryophytes to two members in basal angiosperms. In turn, the RLCK XI subfamily increased in number in monocots and dicots, with 2-3 members in monocots, and up to 8 members in the genome of dicot *Kalanchoe laxiflora* (**Figure 2**). Interestingly, AthRLCK XI-1 was grouped separately from the other AthRLCK XI members in clade 2. RLCK XI members from the same monocot and dicot species could be found in both clades, but RLCK XI members from gymnosperms, ferns, lycophytes, and bryophytes from the same species were exclusively found in one clade but not both. We did not detect RLCK XI members in the chlorophytes, such as *Chlamydomonas reinhardtii*, *Dunaliella salina*, *Volvox carteri*, *Coccomyxa subellipsoidea*, *Micromonas pusilla*, and *Ostreococcus lucimarinus*. Our results show that the RLCK XI subfamily is conserved in land plants. The presence of RLCK XI among land plants suggest that they may play a crucial role in the evolution of the land plant lineage.

To test whether the RLCK XI-KID influenced phylogenetic relationships, we performed the same procedure using only their KID sequences (**Supplementary Figure 2**). The constructed RLCK XI-KID tree demonstrated similarities to the full-length tree. The RLCK-KID tree remained split into two major clades where lineages and plant species were grouped similarly to the full-length tree. RLCK XI members from monocots and dicots clustered similarly in the RLCK XI-KID tree. However, RLCK XI from *M. polymorpha*, *P. patens*, and *S. fallax* became a sister of ferns, gymnosperms, and angiosperms in clade 1 (**Supplementary Figure 2**). These results suggest that the RLCK XI-KID is a conserved region among members of RLCK XI.

Additionally, we performed a BLAST search using the KID of AthRLCK XI-2 to examine whether the RLCK XI-KID is present in sequences other than RLCK XI. A total of 1346 hits that included both hypothetical and predicted proteins were identified and all of them could be traced as members of the RLCK XI subfamily based on their sequence length and coverage of the AthRLCK XI-2 KID (**Supplementary Table 4**). These results show that the RLCK XI-KID is exclusively found in members of RLCK XI within the plant lineage.

### The RLCK XI-KID contains intrinsically disordered regions and conserved sequence motifs

We observed that the length of the RLCK XI-KID has been dynamic in the course of land plant evolution, ranging from 199-527 amino acids, with 280 amino acids on average. The longest KID belonged to MpoRLCK XI-1, which is the only RLCK XI homolog found in *M. polymorpha* (**Supplementary Table 2**).

**Figure 3:**
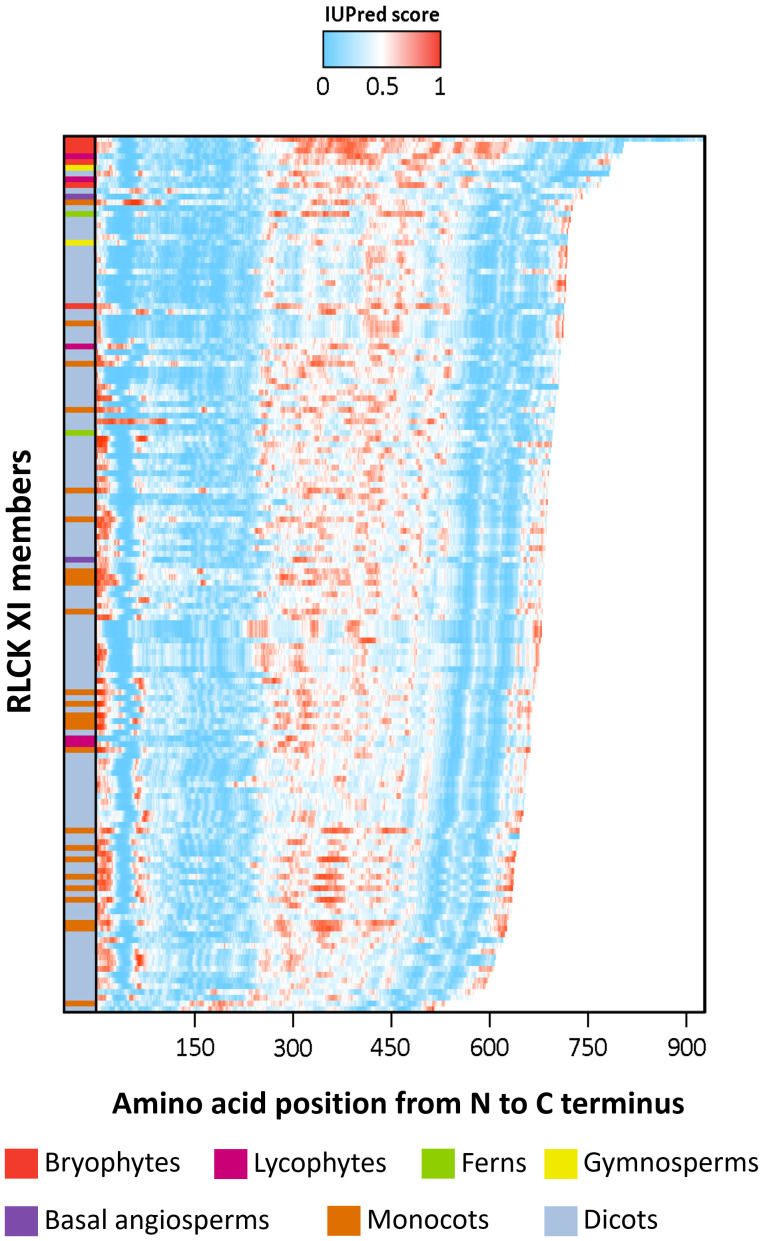
The intrinsically disordered region (IDR) of RLCK XI members corresponds to the kinase insertion domain. Energy estimation of full-length RLCK XI sequences based on IUPred2A. The color gradient represents the propensity of a protein region to be disordered, with darker shades of blue indicating lower disorder and darker shades of red indicating higher disorder. RLCK XI members are arranged according to their sequence length. RLCK XI members from different species are indicated by different colors in the left column.

Previous studies have shown that the activation segments of protein kinases function as flexible contact sites with high intrinsic disorder relative to the surrounding protein kinase core (Gogl et al., 2019). We thus examined whether the RLCK XI-KID also exhibits this property. The full-length sequences of RLCK XI members were used to predict the distribution of IDRs through IUPred2A (Dosztanyi et al., 2005). Based on the propensity for disordered regions, the kinase domain-containing conserved subdomains I-VII and VII-XI have a low propensity for being disordered. In contrast, the KIDs clearly show a high tendency for disorder among most RLCK XI members (**Figure 3**). This result reveals that, like the activation segment of protein kinases, RLCK XI-KIDs also presented qualities found in IDRs.

**Figure 4:**
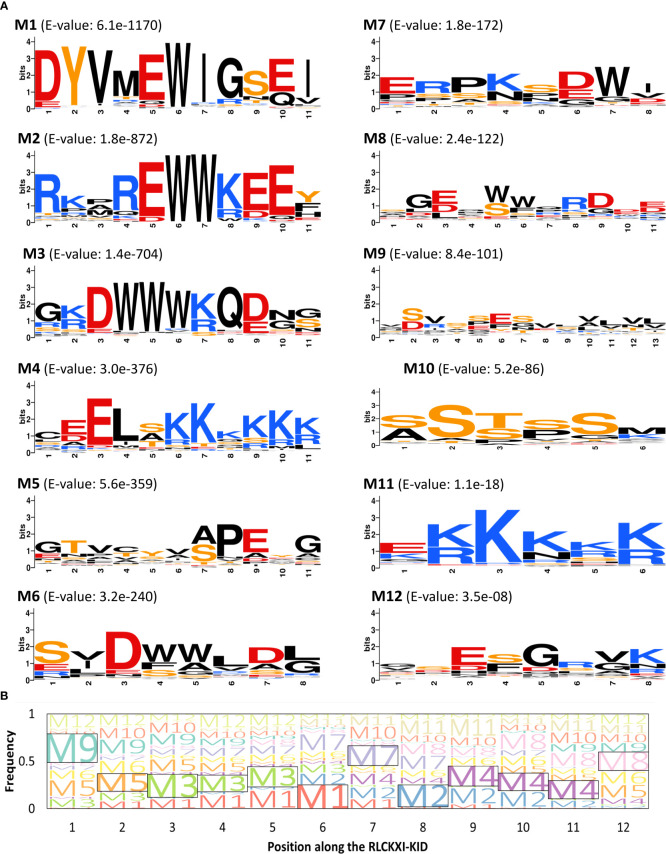
Members of RLCK XI contain conserved motifs in their kinase insert domain. **(A)** Conserved motifs in the RLCKXI-KID were arranged according to increasing E-value (M1-M12). The amino acids are color-coded as follows: acidic residues (D, E), red; basic residues (K, R), blue; polar residues (S, T, Y), orange; others, black. Sequence logos were built using WebLogo. **(B)** Position of conserved motifs along the kinase insert domain of RLCK XI. The frequencies of each of the twelve motifs (M1-M12) were mapped along twelve positions (P1-P12). The motif with the highest frequency in each of the twelve positions is enclosed in a box.

Next, we wanted to know whether conserved motifs existed within the RLCK XI-KID. We observed that the 152 RLCK XI-KID sequences we collected had an average sequence identity of 40%. We examined these sequences for ungapped sequence motifs using the Multiple Em for Motif Elicitation (MEME) suite (http://meme-suite.org/) (Bailey et al., 2015) as a means to determine the origin of the observed sequence similarity. We found twelve conserved motifs with E-values of less than 0.01 across all RLCK XI-KID sequences (**Figure 4A**). These motifs were numbered from lowest E-value (M1) to highest (M12). Interestingly, sub-motif D/EW and D/EWW variations were observed in motifs M2, M3, and M6, while motifs with polylysine residues were detected in M4 and M11. We used the D/EWW-containing M2 and M3 sequences of AthRLCK XI-2 to perform a BLAST search against the NCBI non-redundant protein database to determine whether these motifs could be found outside of RLCK XI. We only found M2 or M3-containing sequences belonging to members of RLCK XI as determined by sequence homology search (**Supplementary Tables 5**, **6**). These results suggest that the conserved motifs within the KID are unique to members of RLCK XI.

In our motif search, we observed that each of the 12 motifs identified only occurred once in all 152 RLCK XI-KID sequences. We wanted to investigate whether these motifs were positioned at random or arranged in a discernable pattern along the RLCK XI-KID. We divided the RLCK XI-KID into twelve positions (P1-P12) based on the single occurrence of each of the twelve motifs in all RLCK XI-KID sequences. If a discernable pattern exists in the KID, then each position would reflect a high frequency of specific motifs in that position. When we explored the position or arrangement of these motifs (**Figure 4B**), we found that M8 and M9 were positioned most frequently at the N- and C-terminal ends of the RLCK XI-KID. Interestingly, M3 and M4 had the highest frequency in multiple positions. M3 had the highest frequency in P3, P4, and P5. Similarly, M4 had the highest frequency in P9, P10, and P11. We noticed a prevalence of charged residues in M1, M2, M3, M4, and M11. On the other hand, M10 was composed of mostly serine residues. M10, M11, and M12 were found across multiple positions on the KID. We hypothesize that the prevalence of M10, M11, and M12 was in part due to the short sequence that made up their respective motifs. These results show that conserved motifs within the RLCK XI-KID have distinct sequence and spatial properties. The conservation of these motifs implies the involvement of potential functions.

### The KID of RLCK XI members possesses nuclear localization signals

Disordered regions have been shown to contain a high frequency of interfaces for protein-protein interaction. Some of these sites exhibit both regulatory and signaling functions and are referred to as eukaryotic linear motifs (ELMs) (Kumar et al., 2022). ELMs are evolutionarily malleable sequences and interact with relatively low affinity due to the limited number of residues that make direct contact with the binding partner (Kumar et al., 2022). These features confer ELMs with the ability to mediate transient interactions to maintain robust cell signaling (Davey et al., 2012; Kumar et al., 2022).

**Figure 5:**
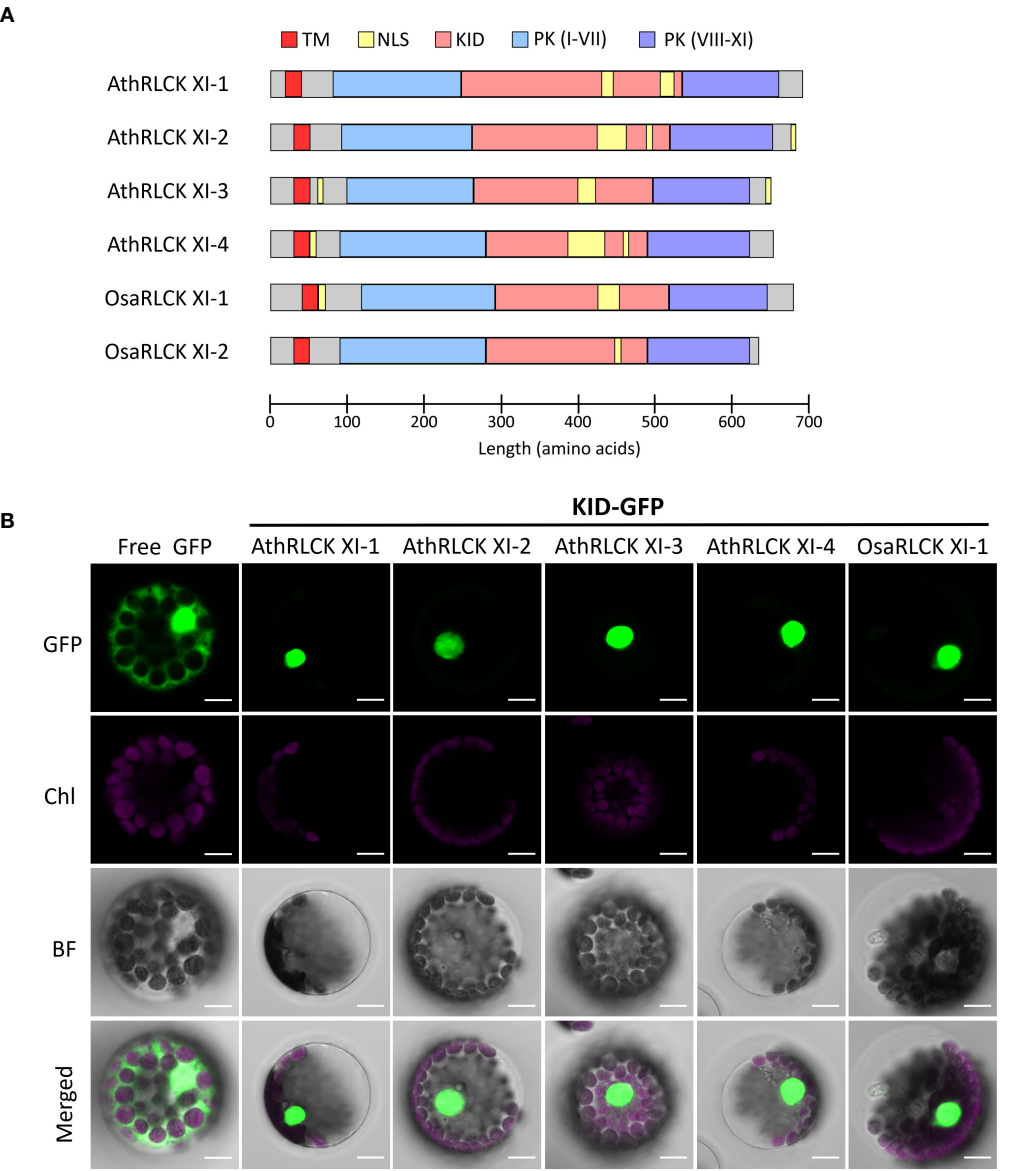
Subcellular localization of the KID from different RLCK XI members. **(A)** Protein domain model of RLCK XI members from *A*. *thaliana* and *O. sativa*. The transmembrane domain (TM), protein kinase (PK) domain, KID and repetitive NLS are indicated. The protein kinase domain for RLCK XI sequences was annotated from the Conserved Domain Database (CDD). Nuclear localization signals were predicted using the Eukaryotic Linear Motif (ELM) resource. **(B)** Subcellular localization of GFP-tagged KID from different RLCK XI members expressed in Arabidopsis protoplasts. The fluorescence signals of GFP and chloroplasts (Chl) are merged on the bright field (BF) images. Scale bars: 5 µm.

To explore the functional relevance of RLCK XI-KID, we searched the ELM resources (Kumar et al., 2022) to examine whether the KIDs of four Arabidopsis RLCK XI members contain any ELMs. We identified several ELMs, annotated as “TRG_NLS_Bipartite_1”, “TRG_NLS_MonoCore_2”, “TRG_NLS_MonoExtC_3” or “TRG_NLS_MonoExtN_4” (**Supplementary Table 7**), which belong to bipartite variants of the classical basic and charged nuclear localization signal (NLS). Interestingly, these NLSs repeatedly occur in the KID (**Figure 5A**) and overlap with the conserved motifs M2 and M3, suggesting that the conserved motifs found in RLCK XI members could be functionally relevant. Similarly, the BLAST search for sequences containing the NLS that overlapped M2 and M3 were members of RLCK XI in plants (**Supplementary Table 8**). In addition to the KID, NLS could be identified in the N and/or C termini of several AthRLCK XI members except for AthRLCK XI-1 (**Figure 5A**). Similar to Arabidopsis, two RLCK XI members from rice also contain NLSs in their KIDs.

### Members of AthRLCK XI are dually localized on the plasma membrane and in the nucleus

To examine whether the observed NLSs in the KID of AthRLCK XI members are functional, we tagged the protein with a green fluorescent protein (GFP) at the C-terminus. We performed a transient expression assay in Arabidopsis protoplasts to observe their subcellular localization using confocal microscopy. While the free GFP was distributed in the cytosol and nucleus, we found the GFP-tagged KIDs of all four AthRLCK XI members and one OsaRLCK XI member were strictly confined in the nucleus (**Figure 5**). These results indicate that the predicted NLSs in the KID of RLCK XI are functional in mediating nuclear import.

**Figure 6:**
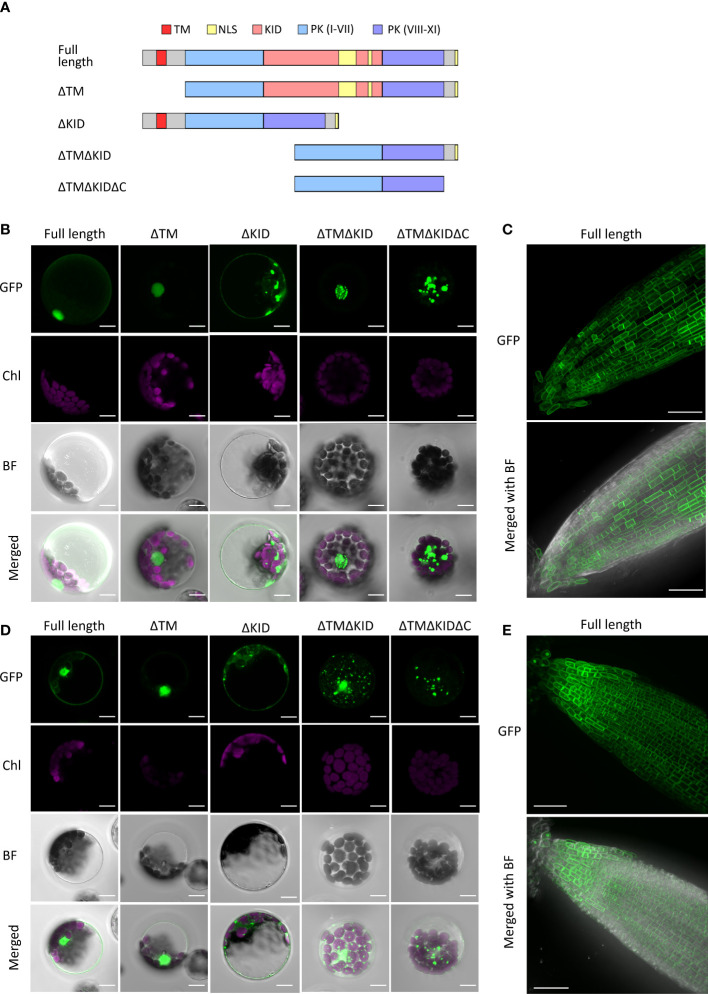
Subcellular localization of AthRLCK XI‐2 and AthRLCK XI‐3. **(A)** Truncation and deletion variants of AthRLCK XI-2 and -3. AthRLCK XI-2 was used as a model to show the different deletion variants used, which include: deleted N-terminal transmembrane domain (ΔTM), deleted kinase insertion domain alone (ΔKID), deleted TM and KID (ΔTMΔKID), and ΔTMΔKID without C-terminal end (ΔTMΔKIDΔC). Subcellular localization of GFP‐tagged AthRLCK XI‐2 **(B)** and AthRLCK XI‐3 **(D)** variants with domain/region deletions expressed in Arabidopsis protoplasts. The fluorescence signals of GFP and chloroplasts (Chl) are merged on the bright field (BF) images. Scale bars: 5 μm. Subcellular localization of estradiol‐induced GFP‐tagged full‐length AthRLCK XI‐2 **(C)** and AthRLCK XI‐3 **(E)** in Arabidopsis root tips. The GFP signals are merged on the bright field images (bottom panels). Scale bars: 40 μm.

The nuclear localization of the KID in AthRLCK XI members was unexpected due to the presence of a single predicted transmembrane domain in all four members. To validate whether RLCK XI members are membrane-bound RLKs, we transiently expressed the full-length AthRLCK XI tagged with GFP at the C-terminus and observed their subcellular localization in Arabidopsis protoplasts. In the following experiments, we only focused on AthRLCK XI-2 and -3 because we were unable to clone the full-length transcripts of AthRLCK XI-1 and -4 due to unknown reasons. Transient expression of full-length AthRLCK XI-2-GFP and -3-GFP revealed their localization on the plasma membrane and in the nucleus with a lesser signal in the cytosol (**Figures 6B, D**). On the other hand, protoplasts expressing AthRLCK XI-2 and -3 without a transmembrane region (ΔTM-GFP) showed localization signals exclusively in the nucleus, similar to the GFP-tagged KID sequence. However, expression of AthRLCK XI-2 and -3 without the KID (ΔKID-GFP) resulted in signals on the plasma membrane and cytosol, where the signals often formed aggregates (**Figures 6B, D**). Intriguingly, expression of AthRLCK XI-2 and -3 that lacked a transmembrane region and KID (ΔTMΔKID-GFP) resulted in punctate signals in the nucleus (AthRLCK XI-2) or the cytosol with less uniform nuclear signals (AthRLCK XI-3) (**Figures 6B, D**). We presume this nuclear localization may result from the predicted NLS sequences in the C-terminal region of AthRLCK XI-2 and -3. Indeed, further removal of the C-terminus (ΔTMΔKIDΔC-GFP) resulted in punctate signals in the cytosol and none in the nucleus (**Figure 6A**). These results suggest KID is essential for the proper nuclear localization of AthRLCK XI-2 and -3. Consistent with the results observed in the protoplasts, full-length AthRLCK XI-2 and -3 mainly localized on the plasma membrane and in the nucleus of transgenic Arabidopsis root tips when induced by β-estradiol (**Figures 6C, E**).

The observation of dual localization of full-length AthRLCK XI-2 and -3 on the plasma membrane and nucleus raises a possibility that the plasma membrane-anchored N-terminal portions of AthRLCK XI-2 and -3 may be proteolytically cleaved, triggered by an unknown signal. As a result, the transmembrane domain-truncated C-terminal portion of AthRLCK XI is redirected to the nucleus *via* the NLS signals in the KID to transmit and relay the signal. It is worth noting that although NLS sequences could be found in the KID and the C-terminal end of AthRLCK XI-2 and -3, the C-terminal NLS sequences alone were insufficient to mediate the nuclear localization observed in full-length AthRLCK XI-2 and -3 (**Figure 6A**).

## Discussion

Receptor-like kinases perceive and transduce extracellular signals into downstream cascades that allow plants to respond to biotic and abiotic stresses, as well as developmental cues (Jose et al., 2020). Much of the diversity in signal perception associated with RLKs is due to their extracellular domain, which can bind to an equally large array of peptides and ligands (Hirakawa and Sawa, 2019; Zhou et al., 2019). In contrast, little is known about the possible structural variations in their protein kinase domain (Bradley et al., 2021). The protein kinase domain contains well-characterized lobes, regions, and subdomains that facilitate the proper folding of its protein structure and catalytic activity (Modi and Dunbrack, 2019). Compared to the otherwise stable structure of the N-terminal and C-terminal lobes, the activation segment found in the C lobe exhibits the property of IDRs and is essential for functional activation (Taylor et al., 2019; Dyla and Kjaergaard, 2020). In this study, we characterize a unique subfamily of RLK, RCLK XI, whose members possess an unusually long activation segment within the kinase domain, designated as KIDs. Several lines of evidence characterize the RLCK XI-KID as an IDR containing functional nuclear localization signals wedged within their protein kinase domain. RLCK XI members present an evolutionarily conserved family in land plants with a unique protein kinase domain architecture.

### RLCK XI is a novel model for a KID-containing protein kinase in land plants

Members of the RLCK XI subfamily from *A. thaliana* have been classified in previous literature as part of large-scale phylogenetic studies aiming to place RLKs as a whole in the protein kinase superfamily (Shiu and Bleecker, 2001; Shiu and Bleecker, 2003; Lehti-Shiu et al., 2009). However, to our knowledge, there has been no specific report of a KID in any of them. Full-length protein alignment of representative protein kinases alongside members of RLCK XI clearly shows the presence of the KID with an average length of 280 amino acids that splits the conserved subdomains I-VII from subdomains VIII-XI (**Figure 1** and **Supplementary Figure 1**). In protein kinases, this region corresponds to the activation segment, which is 20-35 amino acids long and is less conserved within the kinase domain (Nolen et al., 2004). The activation segment, usually exhibiting qualities similar to IDRs, functions as a site for domain interaction and for regulating kinase activity (Taylor and Kornev, 2011; Gogl et al., 2019). KIDs were previously described as anomalous sequences exhibiting qualities present in IDRs with little sequence similarity between protein kinase domains of RTKs (Locascio and Donoghue, 2013; Koike et al., 2020). Although RLCK XI KIDs exhibited properties similar to IDRs (**Figure 3**), the presence of several conserved motifs that overlapped with predicted NLS within this region suggests that the RLCK XI KID has a role in RLCK XI function (**Figure 4** and **Supplementary Table S8**). Indeed, classical NLS have often been found in IDRs of cargo proteins (Wubben et al., 2020). Furthermore, KIDs found in RTKs contained phosphorylation sites that regulated protein function (Locascio and Donoghue, 2013).

It would be worth exploring whether the KID affects RLCK XI protein kinase activity. To conduct the *in vitro* kinase activity assay, we attempted to produce recombinant proteins of AthRLCK XI-2 and AthRLCK XI-3 without the N-terminal transmembrane domain (ΔTM) by expressing them in *E. coli.* Unfortunately, the recombinant proteins were not expressed for unknown reasons. We suspect the intrinsically disordered property of the KID may hinder their expression in *E. coli*. Nevertheless, we could glean some insight from previous studies. First, we’ve observed that members of AthRLCK XI have all the subdomains (from subdomains I to XI) found in functional protein kinases. In contrast, pseudokinases have been shown to lack one or more of these subdomains in their protein sequence (Kannan and Taylor, 2008; McClendon et al., 2014; Kwon et al., 2019). Second, the location of the KID corresponds to the activation segment, which contains regulatory regions and phosphorylation sites that regulate protein kinase activity (Nolen et al., 2004; Taylor et al., 2012). We hypothesize that the RLCK XI-KID has the potential to influence protein kinase activity due to being in the same region as the activation segment in other protein kinases. Interestingly, members of RLCK XI are also classified as non-RD protein kinases. This property refers to protein kinase subdomain VI, which follows the HxD motif wherein x could be R or another amino acid (Kornev et al., 2006). RD protein kinases have been shown to require autophosphorylation on the activation loop for regulation (Adams, 2003; Steichen et al., 2010). In contrast, non-RD protein kinases can be regulated through mechanisms other than autophosphorylation (Dardick et al., 2012; Bender et al., 2021). The presence of the KID and the non-RD subdomain VI in RLCK XI may indicate that these protein kinases are regulated through an alternate mechanism from phosphorylation within the activation segment.

The RLK family of protein kinases has expanded in land plants, which resulted in the various subfamilies observed in present-day plant species (Shiu and Bleecker, 2003; Lehti-Shiu and Shiu, 2012; Dievart et al., 2020). Recent studies have identified sequences belonging to the RLK family in glaucophytes (Gong and Han, 2021). However, we have only traced members of RLCK XI up until bryophytes. The conservation and small number of the RLCK XI subfamily is similar to previously described RLK subfamilies found in plants (Lehti-Shiu and Shiu, 2012). However, none of these other RLK subfamilies have the KID (**Supplementary Figure 1**). Having identified the unique domain architecture in members of RLCK XI, we speculate that during an event in the course of land plant evolution, likely aquatic-to-land plant transition, this RLK acquired the KID we observe today. How the ancestral RLK acquired the KID requires further study. Nevertheless, their conservation among land plants suggests they may have played a role in land plant evolution.

### Functional implication of RLCK XI

A single-pass transmembrane region in the AthRLCK XI sequences suggested their localization on the plasma membrane (**Figure 5A**). However, detecting multiple NLSs within the RLCK XI-KID, the juxtamembrane region, and their C-terminal sequence also suggested their nuclear localization (**Figure 5A**). Different truncated variants of RLCK XI proteins (**Figure 6A**) proved that the NLS were functional in directing their nuclear localization. The dual localization of full-length AthRLCK XI-2 and -3 on the plasma membrane and in the nucleus further verified the functionality of both the transmembrane region and NLS (**Figures 6B–E**). We attempted to tag either the N-terminus or C-terminus of full-length AthRLCK XI-2 and AthRLCK XI-3. However, the N-terminal GFP-tagged protein resulted in irregular aggregates in the cytosol of protoplasts. We speculate that N-terminal GFP tagging of AthRLCK XI disrupt their targeting on the plasma membrane. We, therefore, only showed the result of the C-terminal GFP-tagged proteins. In the future, adding a different fluorescent protein tag downstream of the transmembrane domain but upstream of the kinase domain of the C-terminal GFP-tagged AthRLCK XI may allow us to track changes in their subcellular localization.

The dual localization of AthRLCK XI-2 and -3 on the plasma membrane and in the nucleus suggests that these proteins may undergo proteolytic cleavage before translocating to the nucleus. Indeed, proteolytic cleavage of plant RLKs to release the intracellular fragment has been reported. In rice, the LRR-RLK Xa21 confers broad-spectrum resistance to *Xanthomonas oryzae pv. Oryzae* (Song et al., 1995). Xa21 undergoes proteolytic cleavage, leading to the translocation of its intracellular kinase domain to the nucleus. Xa21 has been shown to bind WRKY62 in the nucleus of rice protoplasts (Peng et al., 2008; Park and Ronald, 2012). Several RLCK members were also shown to undergo translocation from plasma membranes to the nucleus as a part of their signaling cascade. BIK1 is translocated to the nucleus and interacts with WRKY transcription factors that regulate Jasmonic acid and salicylic acid levels (Lal et al., 2018). Similarly, chitin perception-induced PBS1-LIKE 19 (PBL19) can be translocated to the nucleus, where it interacts with WRKY8 to regulate its own transcription (Li et al., 2022). In another case, the RLCK *Puccinia striiformis*-Induced Protein Kinase 1 (PsIPK1) found in wheat interacts with the fungal effector PsSpg1 from *Puccinia striiformis* to promote its nuclear localization to phosphorylate CCAAT-binding transcription factor 1d (TaCBF1d) and enhance susceptibility to the pathogen (Wang et al., 2022). Since RLKs are widely recognized as receptors and mediators for plant development and stress response, we speculate that upon being triggered by a specific signal, RLCK XI may be cleaved by a protease and undergo translocation from the plasma membrane to the nucleus. RLCK XI may function to transmit the extracellular signal to the nucleus for a signaling cascade. The signal and protease responsible for inducing this translocation mechanism have yet to be determined.

It would be fascinating to know the physiological role of RLCK XI members. Unfortunately, to the best of our knowledge, there have been no prior studies on them. According to the publicly available expression profiles (Arabidopsis RNA-seq database, http://ipf.sustech.edu.cn) (Zhang et al., 2020), all the members of AthRLCK XI were expressed ubiquitously except in the pollen (**Supplementary Figures 3A–D**). Members of AthRLCK XI were also expressed under different biotic stresses with some variations (**Supplementary Figures 3E–H**). We attempted to characterize the T-DNA insertion Arabidopsis mutants of all four AthRLCK XI members individually, but none of the single mutants showed developmental defects. Higher-order mutants or specific stress conditions may be employed to examine their function in the future. On the other hand, the transgenic Arabidopsis lines with inducible expression of AthRLCK XI (**Figures 6C, E**) will be excellent materials for functional characterization in the future.

In summary, although the function of the RLCK XI members remains elusive, our characterization of RLCK XI in this study has expanded on the structural diversity known for the protein kinase domain and the assortment of evolutionary changes undergone by receptor-like kinases in plants.

## Data availability statement

The datasets presented in this study can be found in online repositories. The names of the repository/repositories and accession number(s) can be found in the article/**Supplementary Material**.

## Author contributions

T-JC conceived and supervised the experiments. CC advised on the project. JY, C-MS and S-FC performed the experiments and analyzed data. JY, CC and T-JC wrote the manuscript. All authors contributed to the article and approved the submitted version.
