# Supplementary material for: Conservation of land plant-specific receptor-like cytoplasmic kinase subfamily XI possessing a unique kinase insert domain
Source: Front Plant Sci. 2023 Feb 22;14:1117059. doi: 10.3389/fpls.2023.1117059 (PMC9992409; doi:10.3389/fpls.2023.1117059)
Supplement: Supplementary file 1 [file DataSheet_1.pdf]

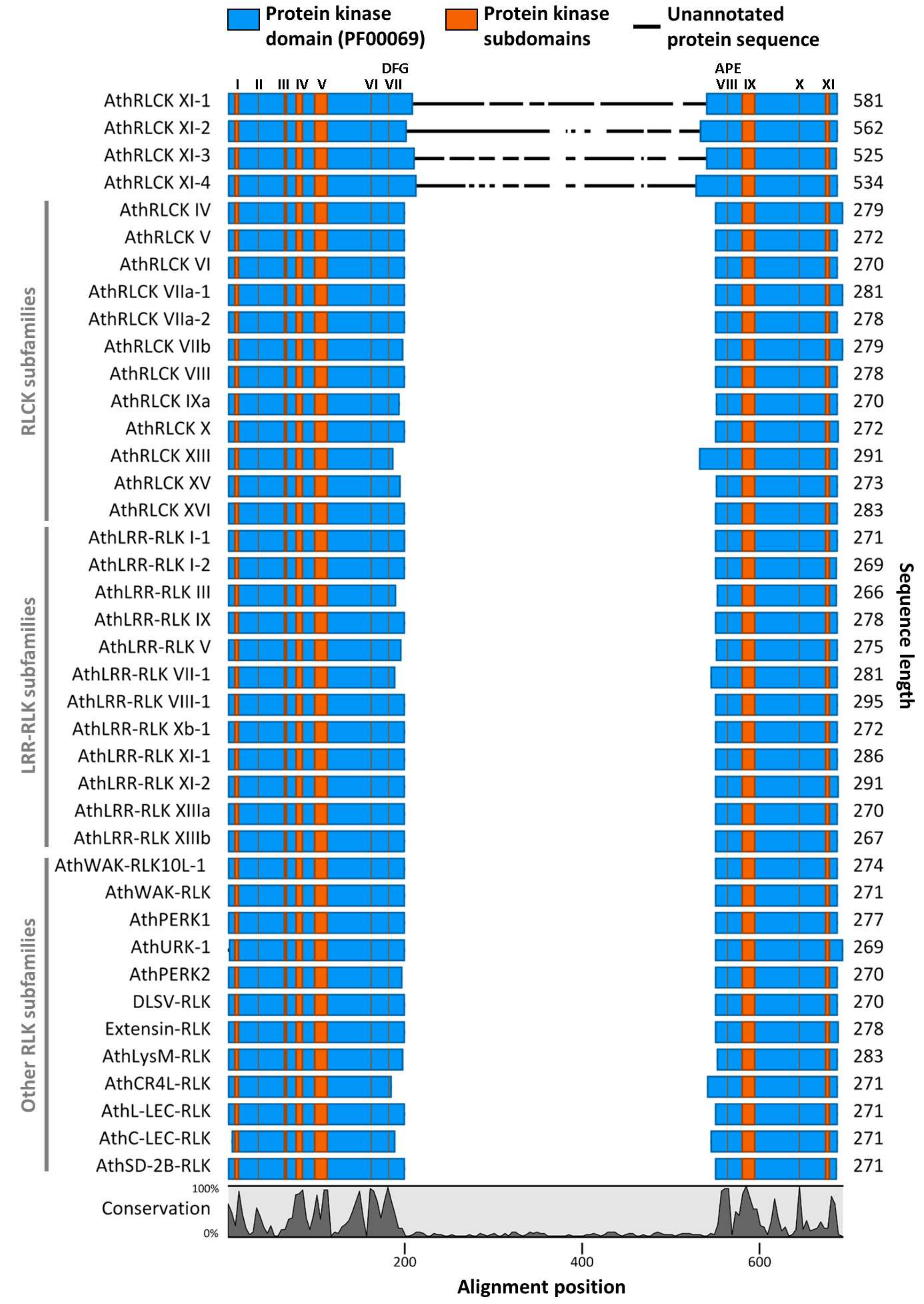

Supplementary Figure 1. Members of RLCK XI from *A. thaliana* possess a kinase insert domain not found in other RLK subfamilies.

Sequence alignment of the protein kinase domains of RLCK XI members, AthRLCK XI-1 (AT1G80870), AthRLCK XI-2 (AT2G45590), AthRLCK XI-3 (AT4G25390), AthRLCK XI-4 (AT5G51770), together with representative RLKs found in *A. thaliana*. The representative RLKs were categorized as RLCK, LRR-RLK, and other subfamilies. Each representative was chosen based on which subfamily member had the highest pairwise alignment score with AthRLCK XI-2. For RLCKs, AthRLCK IV (AT4G00330.1), AthRLCK V (AT1G09440.1), AthRLCK VI (AT2G18890.1), AthRLCK VIIa-1 (AT5G18610.1), AthRLCK VIIa-2 (AT2G26290.1), AthRLCK VIIb (AT1G24030.1), AthRLCK VIII (AT3G59350.1), AthRLCK IXa (AT3G26700.1), AthRLCK X (AT2G25220.1), AthRLCK XIII (AT1G33260.1), AthRLCK XV (AT3G15890.1), AthRLCK XVI (AT2G28250.1). For LRR-RLKs, AthLRR-RLK I-1 (AT3G46330.1), AthLRR-RLK I-1 (AT1G51810.1), AthLRR-RLK III (AT5G16590.1), AthLRR-RLK IX (AT1G66150.1), AthLRR-RLK V (AT4G22130.1), AthLRR-RLK VII-1 (AT1G12460.1), AthLRR-RLK VIII-1 (AT3G53590.1), AthLRR-RLK Xb-1 (AT2G02220.1), AthLRR-RLK XI-1 (AT1G09970.1), AthLRR-RLK XI-2 (AT2G31880.1), AthLRR-RLK XIIIa (AT1G78530.1), AthLRR-RLK XIIIb (AT5G07180.1). Other RLK subfamilies included AthWAK-RLK10L-1 (AT1G25390.1), AthWAK-RLK (AT1G21230.1), AthPERK1 (AT4G32710.1), AthPERK2 (AT3G13690.1), AthDLSV-RLK (AT4G23240.1), Extensin-RLK (AT3G58690.1), AthLysM-RLK (AT1G51940.1), CR4L-RLK (AT3G51990.1), AthL-LEC-RLK (AT5G01550.1), AthC-LEC-RLK (AT1G52310.1), AthSD-2B-RLK (AT5G24080.1). The location of conserved protein kinase subdomains (I-XI) on each sequence (orange) was indicated based on sequence alignment with well-characterized protein kinase sequences. An insertion domain in the RLCK XI members was identified between the conserved motifs Asp-Phe-Gly (DFG) and Ala-Pro-Glu (APE) of subdomain VII and subdomain VIII, respectively.

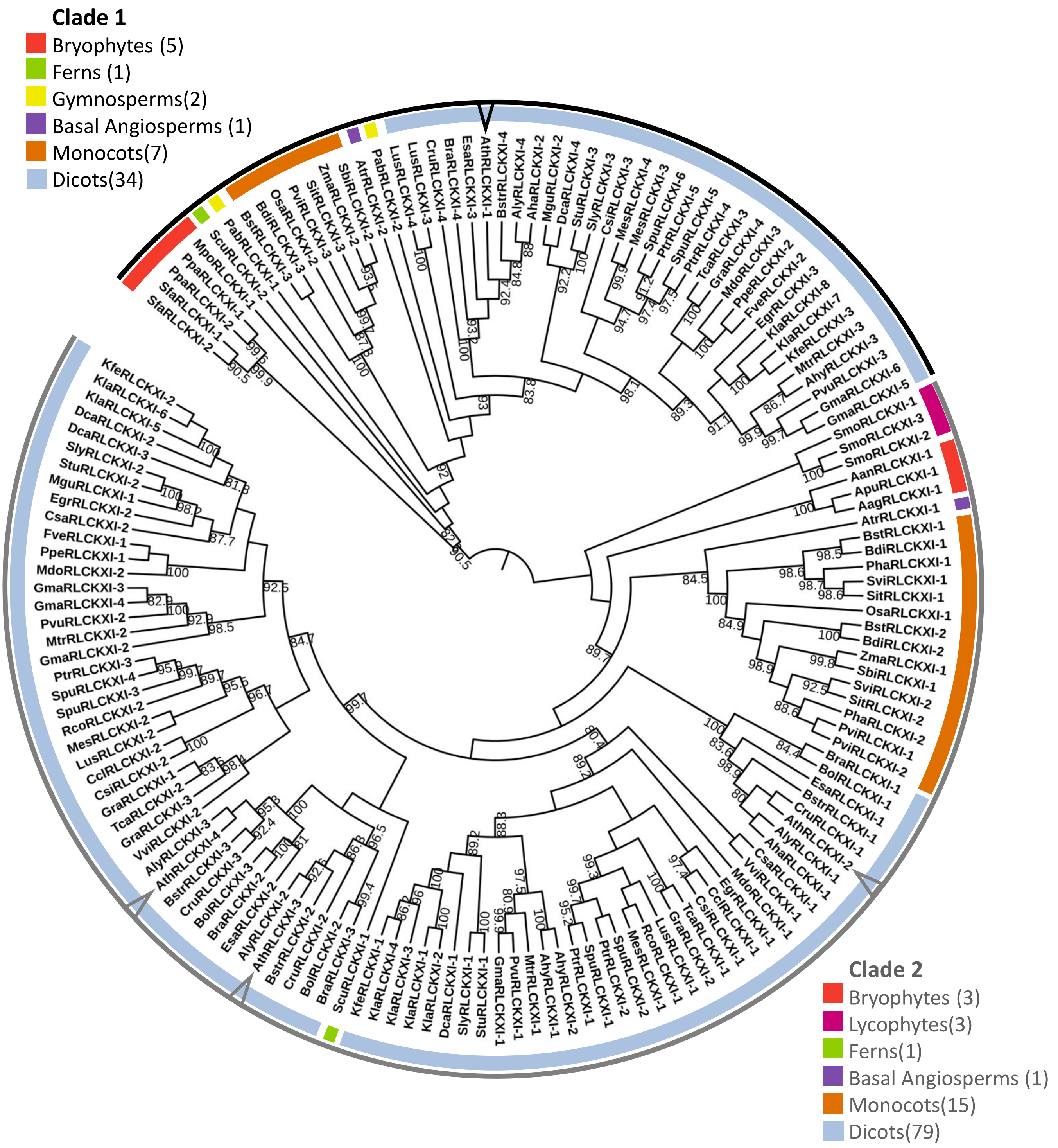

Supplementary Figure 2. The KID of RLCK XI is conserved in land plants.

The phylogenetic tree was constructed based on the protein sequence alignment of the KID of RLCK XI members. The alignment was used as input for IQ-TREE with the maximum-likelihood method. The clades are divided into two major clades (Clades 1 and 2), each containing members from different plant lineages represented by different colors. Members from *A. thaliana* are pointed at in the cladogram. Bootstrap replicates were set to 1000. Nodes showing bootstrap confidence of 80 or higher are shown. Visualization and annotation were conducted using iTOL.

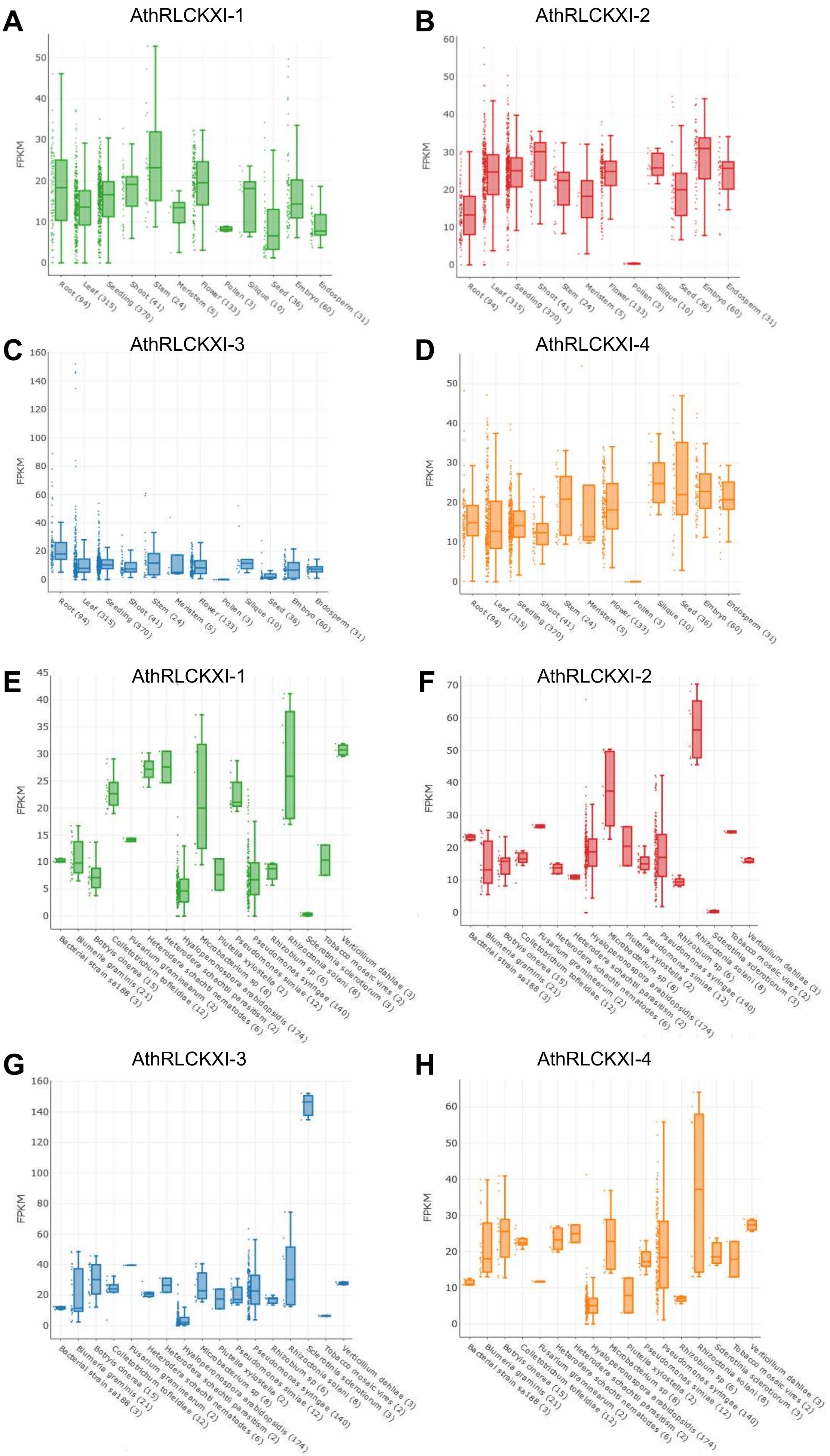

Supplementary Figure 3. Members of AthRLCKXI are ubiquitously expressed in plant tissues and under biotic stress.

Expression profiles of AthRLCK XI members in different tissues (A-D) and under biotic stress (E-H). The expression level is presented as Fragments Per Kilobase per Million (FPKM). The number inside the parenthesis on the x-axis indicates the number of datasets for each tissue or biotic stress condition. Data and experimental parameters were retrieved from the Arabidopsis RNA-seq (ARS) database (http://ipf.sustech.edu.cn).
